# Tele-health interventions to support self-management in adults with rheumatoid arthritis: a systematic review

**DOI:** 10.1007/s00296-021-04907-2

**Published:** 2021-06-16

**Authors:** Alison MacIver, Hannah Hollinger, Clare Carolan

**Affiliations:** 1 Department of Nursing and Midwifery, University of the Highlands and Islands, Inverness, UK; 2 NHS Western Isles, Stornoway, Scotland UK

**Keywords:** Rheumatoid arthritis, Self-management, Tele-health interventions

## Abstract

Rheumatoid arthritis (RA), a long-term auto-immune condition is a challenging condition for patients to manage. Goals of treatment include reducing pain, decreasing inflammation, and improving an individual’s overall function. Increasingly technology is being utilised to support patients to self-manage their condition. The aim of this systematic narrative review was to synthesise and critically appraise published evidence concerning the effectiveness of tele-health interventions to support self-management in RA. Bibliographic databases searched from 2014 to March 2020 included MedLINE, Embase, Cochrane Library. Search strategy combined the following concepts: (1) rheumatoid arthritis, (2) tele-health interventions, and (3) self-management. Only randomised controlled trials (RCTs) involving adults with RA were included. Titles, abstracts, full-text articles were screened, any discrepancies were checked by a second reviewer. Risk of bias was assessed using Cochrane risk of bias tool and data were extracted utilising the Cochrane data collection form for RCT interventions along with the TiDier checklist. Due to high heterogeneity, results were not meta-analysed and instead data were synthesised narratively. The search identified 98 articles, seven were included. The completed RCTs varied in the nature of the interventions, duration/severity of RA, outcomes measured and effectiveness of the interventions. The completed RCTs included a total of 791 participants Disease duration was largely between 4 and 10 years and disease severity on average was moderate. There was extensive variation in intervention components, theories underpinning theories and outcomes measured. Five RCTs reported a positive effect on factors such as disease activity, medication adherence, physical activity and self-efficacy levels. This study suggests that tele-health interventions that are well-designed, tailored and multi-faceted can help to achieve positive self-management outcomes in RA. None of the studies showed evidence of harm.

**Supplementary Information:**

The online version contains supplementary material available at 10.1007/s00296-021-04907-2.

## Introduction

Rheumatoid arthritis (RA) is the most commonly diagnosed systemic inflammatory arthritis [1]. RA is a painful long-term condition which results in wide spread systemic inflammation, and joint damage [2]. Women, smokers, and those who have a family history of the disease are at highest risk of developing the condition [3]. RA affects 0·5–1·0% of adults, with 5–50 per 100,000 new cases annually in industrialised countries [4]. Uncontrolled active RA can result in erosive joint damage, increasing disability, poor quality of life, and other co-morbidities [5]. Patients with RA face a high disease burden including symptoms such as pain, stiffness, fatigue and decreased muscle strength which makes activities of daily living challenging. Furthermore, RA has been linked to psychological issues such as depression, helplessness and anxiety which further impacts on everyday life [6].

Treatment advances in RA have led to substantial improvements in patients’ physical and psychological outcomes, however, issues regarding fatigue, pain, reduced physical activity and quality of life still exist [7]. Poor outcomes are associated with non-adherence to medication, lack of knowledge about the condition and lack of support in coping and effective self-management [8]. Self-management is defined as the ability to manage symptoms, treatment and lifestyle changes which are associated with living with a chronic condition [3]. Evidence shows promoting self-management can contribute to better treatment and health outcomes through addressing these issues [9]. Engaging in self-management supports patients to take responsibility for improving their health by engaging in positive health behaviours such as physical activity, fatigue management and medication adherence [10].

There is potential for tele-health interventions (i.e. interventions delivered via digital technologies such as mobile phones, computers, text messaging) to provide cost effective, safe health care. The World Health Organisation (WHO) promotes the use of tele-health to provide clinical services [8]. The European League Against Rheumatism (EULAR) also recognise the benefits of tele-health for RA patients to enhance patient engagement and self-management approaches in rheumatic diseases [7]. Tele-health interventions can support self-management in RA by using technology to provide patients the knowledge, skills and support to manage their condition [7]. Research shows that patients in remission who are familiar with modern technology would welcome the possibility of managing their own condition by using online devices or mobile phones [11].

Although the use of tele-health interventions for RA is increasingly advocated, systematic reviews to date have only focussed on the quality and features of tele-health interventions available and their potential to be used within RA [11, 12]. These studies identified that tele-health interventions increase the emphasis engaging people with RA as active partners in their care and that a demand exists for technology that is accessible, simple to use and can help with the clinical management of the condition. Najm et al. [13] systematic review assessed the content and development of self-management tele-health interventions and endorsed that patients with RA are keen to engage with technology that supports self-management. A further systematic review by McDougall et al. [14] found tele-health interventions to be effective for the diagnosis and management of inflammatory rheumatic diseases but proposed that further studies were required to determine the best uses of tele-health for the management of these conditions. Knudsen et al. [15] qualitative study on tele-health in RA revealed the need for further insight into how tele-health interventions could be developed to increase patients to have an active role in disease control. To date no systematic review has assessed how effective telehealth interventions are at supporting self-management in patients with RA.

The purpose of this review is to answer the question: ‘Are tele-health interventions effective for supporting patients living with RA to self-manage their condition’? The review aims to evaluate a range of tele-health interventions and summarise the existing evidence base for their effectiveness. The aims include undertaking a systematic search and review of the literature in order to determine: (1) the extent to which tele-health interventions are effective in supporting patients to self-manage RA and (2) address implications for research and practice since no known systematic reviews of the effectiveness of tele-health interventions for supporting self-management in RA has previously been done. Examination of the literature for the usefulness and effectiveness of tele-health interventions in RA will provide insight into role that this technology could have to support self-management and improve patient outcomes.

## Methods

### Search strategy

The Preferred Reporting Items for Systematic Reviews & Meta-Analysis (PRISMA) statement and guidelines guided the conduct of this systematic review [16]. The following electronic bibliographic medical databases were systematically searched to identify trials of tele-health interventions supporting self-management in adults with RA: The Cochrane Central Register of Controlled Trials (CENTRAL), Ovid MedLINE, Ovid Embase. The search terms were grouped into four concepts: (1) Rheumatoid Arthritis, (2) self-management, (3) tele-health interventions, and (4) study type. The search was limited to manuscripts published in the English language between 2014 and the present date to reflect developments in the tele-health industry and promote inclusion of contemporaneous studies. As an example, specific search terms undertaken in Ovid MedLINE on 3rd March 2020 is included (See supplementary File 1 for search strategy).

### Inclusion/exclusion criteria

**Fig. 1:**
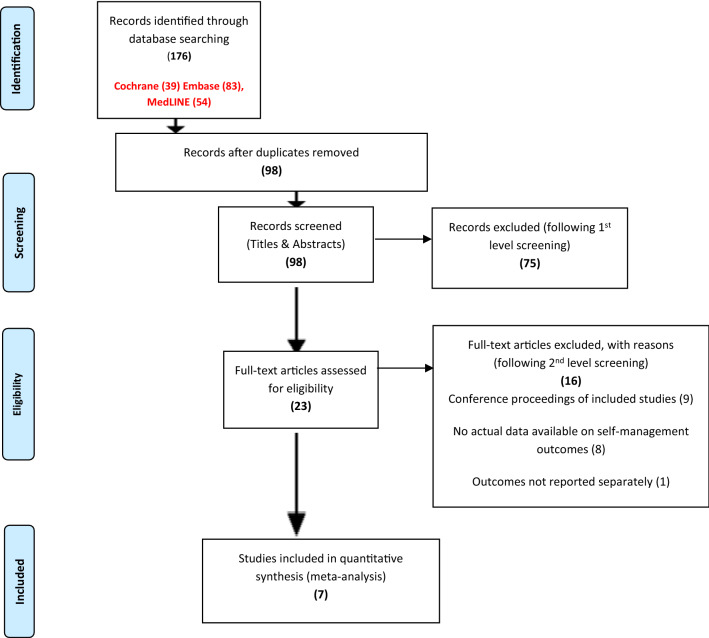
Prisma flow diagram (Moher et al. 2009)

Randomised Controlled Trials (RCTs) were chosen as they measure the effectiveness of an intervention which addresses the research question posed in this review. Articles were included if participants were over the age of 18 and had RA, published in English, the intervention involved tele-heath technology to support self-management and incorporated outcomes of interest. (Table 1). An initial literature search conducted in March 2020 Identified 176 citations which were imported into Refworks and de-duplicated. After removing duplicates 98 articles were identified. First level screening was undertaken independently 98 titles/abstracts were screened and each article was assessed against the pre-set eligibility criteria which was ordered from 1 to 6. Inclusion criteria included language, type of study, participants, type of intervention outcomes and date. Seven studies were selected which met the inclusion criteria. Any discrepancies throughout the process were discussed with other authors (HH, CC) where there was any uncertainty, and the reasons for excluding the studies were recorded. The PRISMA flowchart Fig. 1 demonstrates this process in fuller detail.

**Table 1:** Inclusion/exclusion criteria

	Inclusion	Exclusion
Language	All papers in the English language	Non-English were excluded due to lack of translation facilities
Type of study	Randomised controlled trials (RCTs), controlled non-randomised studies and controlled before and after studies	Qualitative research papers Mixed Method papers incorporating a qualitative/quantitative approach Feasibility, pilot, quasi-experimental studies and conference abstracts
Type of intervention	Tele-health interventions, including any digital intervention accessed through a computer, mobile phone or hand-held device, including web-based or desktop computer programmes or applications that support self-management Comparison groups to the tele-health intervention would be usual care or no intervention	Tele-health interventions that do not support self-management
Type of participants	Adults over the age of 18 of any gender with a diagnosis of RA	Populations incorporating inflammatory arthritis, osteo-arthritis, juvenile idiopathic arthritis, psoriatic arthritis
Type of outcomes	Outcomes of interest include self-management-related areas such as disease activity, including objective and self-reported clinical, physiological markers of disease control. Validated measures of symptoms such as fatigue, pain, disability and quality of life. Further outcomes such as self-efficacy and medication adherence, health care utilisation will also be considered	Subjective measures or generalised outcomes such as patient satisfaction or quality of life
Date	Studies included will be from 2014 onwards to the present date to ensure data are contemporaneous and relevant	Papers prior to 2014 were excluded as tele-health interventions were not so readily available to patients

### Quality assessment

The quality appraisal tool chosen for assessing the quality of studies when undertaking a systematic review adhered to the PRISMA guidelines [16]. The Cochrane Risk of Bias tool was utilised to screen each study for bias [17]. This validated tool is recommended by Cochrane and was chosen for this review as it enables separate assessments of six specific domains of risk, including selection, performance, detection, attrition, and reporting bias. The tool was chosen as it enables a judgement to be made on whether a study has a high, low or unclear risk of bias. Risk of bias was undertaken independently with disagreements resolved by consensus with co-authors (HH, CC) (Table 2).

**Table 2:** Cochrane risk of bias tool [17]

	Selection bias		Performance bias	Detection bias	Attrition bias	Reporting bias	
	Random sequence generation	Allocation concealment	Blinding patients/personnel	Blinding outcome assessment	Incomplete data	Selective reporting	Other biases
Allam et al. [18]	High risk	Low risk	High risk	Unclear	Low risk	Unclear	Unclear
Zuidema et al. [19]	Unclear	Unclear	High risk	Unclear	Low risk	Low risk	Unclear
Kuusalo et al. [24]	High risk	Unclear	High risk	Unclear	High risk	Unclear	Unclear
Song et al. [21]	Unclear	Unclear	High risk	Unclear	Low risk	Unclear	High risk
Liu et al. [22]	Low risk	Low risk	High risk	Unclear	Low risk	Low risk	High risk
Zhao & Chen [23]	Low risk	Low risk	High risk	Unclear	Low risk	Unclear	High risk
Salaffi et al. [20]	High risk	Low risk	High risk	Unclear	Low risk	Unclear	High risk

### Data extraction and analysis

Data extraction were guided by the PRISMA statement [16] to ensure a systematic approach was adopted. Data were extracted using the Cochrane data collection form for intervention reviews: RCTs [2019]. The full-length papers were read, and data extracted, conflicts in data extraction were resolved by consensus with co-authors. Data collected included author, publication year, country of origin, details about the study population (diagnosis, mean age, disease activity, disease duration, and education level), the intervention, comparator, outcomes and study findings. Outcomes of interest related to self-management were grouped into disease control outcomes (clinical and physiological markers of disease control, health care utilisation and validated measures of symptoms) and self-management process outcomes (e.g. self-efficacy, medication adherence). To help determine the effectiveness of tele-health interventions in supporting patients with RA to self-manage their condition; the Template for Intervention Description and Replication (TiDier) checklist [17] was used as an additional data extraction tool. This checklist enabled extraction of further details of the key components of interventions, including identifying the type of technology used to support self-management, characteristics, including intensity, and duration, and underlying theoretical approaches the intervention. This information was then collated into tables to illustrate findings.

### Data synthesis

Due to the extreme heterogeneity of the included studies in terms of participants, location, interventions and outcome measures a meta-analysis was statistically inappropriate and could not be performed. Therefore, a narrative synthesis was undertaken as interventions and associated outcomes were diverse.

## Results

### Outcome of the search

Figure 1 illustrates the flow of studies through the review process and outlines reasons for exclusion. Searches identified 176 potentially relevant articles which was reduced to 98 articles after removing duplicates by critiquing articles against inclusion criteria 1–4 (Table 2). Following review of title and abstracts 23 full text articles were retrieved. Of the 23 full text articles identified seven fully met the inclusion criteria 1–6 and were included in this systematic review.

### Study characteristics and design

Table 3 shows details of each studies setting, participant demographics, interventions, comparator and key findings. The seven studies included in this systematic review dates of publication ranged from 2015 to 2020, six of the studies were undertaken from 2019 onwards reflecting the increasing use of tele-health interventions within healthcare. Each study involved a tele-health intervention and the comparison was usual clinical care. Three studies compared a web based tele-health intervention with usual care [18–20]. Allam et al. [18] trialled a self-management information website with specific groups accessing social support and/or gamification features. Salaffi et al. [20] & Zuidema et al. [19] incorporated self-monitoring tools to track symptoms within their self-management website. Three studies looked at telephone based self-management education sessions as an intervention following hospital discharge compared to usual care [21–23]. One study used a mobile phone text messaging (SMS) application to improve self-management by supporting medication adherence and self-monitoring of symptoms [24]. A wide range of outcomes to measure the effectiveness of interventions were included in the RCTs. The outcome measures were categorised as follows: disease activity and related symptoms, physical activity, knowledge and markers of self-care, medication adherence and health care resource use.

**Table 3:** Study characteri*s*tics & key findings

Author, date, location, design, duration	Aim	Participants	Intervention	Comparator	Outcomes & measurement times	Key findings
Allam et al. [18], Switzerland, RCT, 16 weeks	Examine effect of web-based intervention incorporating online information, social support features and gamification on individuals with RA	5 arm parallel RCT: 155 patients participated (informational website: n = 30; social support: n = 29; gamification: n = 28; control: n = 40) Mean Age 57.95 years Mean duration of RA from diagnosis 11.89 years Disease Activity: Not reported	Group 1: Access to informational website with advice Group 2: Access to informational and social support features Group 3: Access to informational website advice and gaming section Group 4: Access to informational website advice social support and gaming sections	Routine care with no access to the web-based intervention	Physical Activity Exercise Behaviours Scale Health care utilisation scale Prescription medication overuse: Prescription Opioid Misuse Index RA knowledge: Patient Knowledge Questionnaire in RA Empowerment scale Measured at Baseline, 8 & 16 weeks	This study provides evidence demonstrating the potential positive effects of web-based gamification and online social support on health and behavioural self-management in patients with RA
Zuidema et al. [19], Switzerland, Explorative RCT, 12 months	Evaluate efficacy of a web based self-management programme for patients with RA	157 participants I/C: 78/79 Mean age-6 years Disease activity-not reported Average disease duration-not reported	Patients were given 12 months of access to a web-based self-management programme comprising nine modules and a diary to track pain and fatigue symptoms	Routine care with no access to the website	Self-Management Ability Scale (SMAS) Rheumatoid Arthritis Self-Efficacy scale RAND-36 Level of Pain & Fatigue Numerical Rating Scale The Modified Pain Coping Inventory for Fatigue (MPCI-F) The Patient Activation Measurement (PAM-13) Measured at baseline, 24, 52 weeks	No positive effects were found regarding the outcome measurements, effect sizes were low, and results show no statistically significant difference The author questions whether the structure should be modified to determine whether potential benefits could be achieved
Kuusalo et al. [24], Finland, RCT, 12 months	Examine the influence of short messaging service (SMS) text messaging enhanced monitoring to support self-management of early RA	166 participants I/C: 84/82 Mean age-55.5 years Mean Disease Activity Score (DAS) 28–4.60 (moderate disease activity) Mean disease duration-4 years	An SMS text message disease self-management system to enable patients to self-monitor their symptoms in early RA	Usual Care	Boolean Remission C-Reactive Protein levels Utilisation of health care resources scale SF-36 Quality of Life Measured at baseline, 12, 24, 52 weeks	The study failed the primary outcome despite a trend favouring the intervention group with remission rates higher at 6 and 12 months, statistical significance was not reached. Participants found the intervention technology difficult to use The authors concluded that despite a favourable trend in remission, text message enhanced monitoring does not significantly improve RA
Song et al. [21], China, RCT, 12 weeks	Examine effects of a tailored tele-health intervention on medication adherence and disease activity in discharged patients with RA	92 participants: I/C:46/46 Mean age-55.26 Mean disease duration-4.5 years Mean disease activity-DAS 28 = 4.5 (moderate disease activity)	Four tailored self-management educational sessions delivered by telephone by a nurse across a 12-week period following hospital discharge	Usual Care	Medication Adherence Compliance Rheumatology Questionnaire Disease Activity Score (DAS) 28 Measured at baseline, 12 and 24 weeks	This study demonstrated that a tailored tele-health educational intervention could significantly improve patients with RA recently discharged from hospital medication adherence Further research will be required to demonstrate longer-term effects of this intervention
Liu et al. [22], China, RCT, 8 weeks	Examine the effects of a 4-week telephone based transitional care programme to support self-management in patients with RA	88 participants I/C: 44/44 Mean age-49 years Mean disease duration-10 years Mean disease activity not reported	Telephone based self-management education sessions following discharge from hospital to consolidate patient’s self-management behaviours	Usual Care	RA Self Efficacy Score Health Assessment Questionnaire Disability Index (HAQ-DI.) Hospital Readmission Rates Measured at baseline, 4 & 8 weeks	This four-week self-management intervention provided evidence that telephone based self-management sessions following hospital discharge improve self-efficacy. The intervention encouraged patients reduced health care utilisation by reducing hospital readmission
Zhao & Chen [23], China, RCT, 24 weeks	Explore the effectiveness of self-management education programme on self-efficacy in patients with RA	92 participants I/C: 46/ 46 Mean age 55.5-years old Mean disease duration-4 years Mean DAS 28 score-5.05 (indicating high disease activity)	Health education delivered via telephone. Information included exercise, diet and medication following hospital discharge	Participants received one telephone call following	Rheumatoid Arthritis Self Efficacy (RASE) Score HAQ-DI DAS 28 Measured at baseline, 12 and 24 weeks	Self-efficacy of discharged patients with RA improved at week 12 and week 24, however, there was no statistically meaningful difference in disease activity. There was an improvement in DAS 28 scores of the intervention group at week 12 and 24 which could be meaningful
Salaffi et al. [20], Italy, RCT, 12 months	Examine whether a tele-health intervention supports self-management to achieve remission and comprehensive disease control in early RA	41 participants I/C:21/40 Mean age 50 years Mean disease duration 6 months Mean CDAI- 25.7 (high disease activity.)	A web-based programme enabling patients to monitor their own symptoms online and access information. This was monitored by a clinical case manager who could adjust treatment accordingly	Routine Care	CDAI RA Impact of Disease (RAID) Recent Onset of Arthritis Disability (ROAD) score CRP Radiographic evaluative of joint damage Measured at baseline, 12, 24, 36, 52 weeks	Findings support that an intensive treatment strategy utilising a web based tele-health intervention to enable patients to self-monitor their symptoms and access to relevant information promotes self-management

### Quality assessment and risk of bias

Results for the Cochrane Risk of Bias tool [17] for the included RCTs are reported in Table 2. Despite using random sequence generation participants were being selected from a group already identified to have access to either Internet or a mobile phone in several studies indicating a high risk of selection bias [18, 20, 24]. The risk of detection bias was unclear across all studies as it was not certain whether blinded outcome assessment had been performed. One study was assessed to have high risk of attrition bias as the data was incomplete [24]. All studies were unclear or at high risk of bias in most domains, common issues included small numbers of participants and a lack of blinding [20–23]. Other high risk of bias included participation bias as only those who had a telephone or mobile phone were recruited [20–23]. The studies were of low-to-moderate quality affecting overall validity, reliability and generalisability.

### Synthesis of results

#### Intervention characteristics

Theories underpinning each intervention were identified and summarised in the TIDieR intervention Table 4. This mainly outlined cognitive-behavioural approaches, including self-efficacy theory [18, 23], empowerment theory, social support theory [18], theory of planned behaviour [19], dual process theory [19, 21, 23], and health belief theory [21]. A treat to target approach was mentioned as a key component of supporting self-management in two studies [20, 24]. Each intervention was delivered as planned with the overall aim of supporting self-management in individuals with RA.

**Table 4:** Tidier table – please see attachment 1

Author/year	Brief name	Recipients	Why	What (materials & procedures)	Who (provided)	How & where	When & how much	Tailoring	Modification of intervention throughout trial	Extent of intervention fidelity
Allam et al. [18]	ONESELF Website including information, online social support and gamification	Patients > 18 with a diagnosis of RA	Web based intervention based on dual process and empowerment theory to help patient’s better cope and manage their condition by providing accurate information. Planned behaviour change theory was incorporated aiming for behaviour change by imparting the health information required to help them manage their disease Social support theory was incorporated as this is associated with self-management skills Gamification involved the application of game design to motivate by providing rewards to engage with the website	A website designed by medical staff sought to adopt a patient centred approach Sections of the website included information about RA and aimed to improve knowledge particularly in relation to physical activity & medication. Treatment options and disease management strategies An open forum and chat room was provided by clinicians This incorporated a patient blog to contribute and write to each other or ask questions Gamification was added to encourage patients to interact. Participants engagement with the materials earned points Participants received a face to face session of thirty minutes to educate them on how to use the website	Rheumatology health care professionals Social support provided by fellow patients	Delivered over the Internet Participants were given their own account for the website Patients were also sent SMS text messages notifying them about chat room sessions and inviting them to participate	Minimal engagement was requested was for one hour per week	The website was frequently updated to respond to users’ questions, and participants would receive an email outlining new information	Not described	Delivered as planned. The usage of the website was monitored by recording the number of logins to each section of the website On average participants paid a mean 53.68 (SD 93.07) visits to the various sections of ONESELF Groups who were offered the gamified experience used the website more often
Zuidema et al. [19]	Web based self-management enhancing programme	Patients > 18 with a diagnosis of RA	Website to help individuals manage the symptoms of RA The website sought to elicit behavioural change by providing information to promote positive self-management behavioural changes.	The programme was developed in collaboration with RA patients and RA specialist healthcare professionals The Web-based self-management programme comprised 9 modules covering symptom management, diet and exercise and a diary to track patients fatigue and pain Each module comprised 2–5 sessions and assignments Participants received a written instruction manual for the programme	Rheumatology Health care Professionals	Web based Online	No set requirement Participants could engage as and when they wanted too Reminders to visit the programme were sent twice weekly via email Clinicians brought the programme to the attention of participants during consultations	Not described (authors recognised that the ability to tailor the performance objectives may improve the efficacy of the intervention.)	Not described	Intervention was delivered as planned
Kuusalo et al. [24]	Automated Text Message-Enhanced Monitoring	Participants > 18 early stage RA	A text message monitoring system that patients can use to help self-manage their RA by monitoring their own disease activity and medication adherence and sharing that information with health care professionals Intervention is based on the treat to target approach of early RA requiring frequent monitoring and targeted treatment	Showing Any Need for Reassessment (SANDRA) software. Patients in the intervention group were given written and 30-min presentation on performing SMS monitoring Patients received text messages every N fortnight during weeks 0–24, asking patients to report on their disease activity and medication adherence The software included a cut off limit for scores, very low cut off limits were chosen in order to detect possible problems early and to improve chance of reaching early strict remission	Rheumatology Clinicians monitored the responses and gave the patient a telephone call within 48 h if problems were highlighted	SMS text messaging via mobile phone technology Participants were expected to own a mobile phone and be competent in using it	Patients received text messages every fortnight during weeks 0–24	Each response was evaluated, and further clinic appointments triggered if required	Not described	Intervention was delivered as planned 52% found assessing their disease activity difficult
Song et al. [21]	Telephone delivered self-management education	Patients > 18 with RA recently discharged from hospital (following admission for RA related reasons)	This intervention focussed on helping patients perceive the consequences of RA, educating them on medication management skills and empowering them with the knowledge to self-manage their condition	Educational sessions delivered by telephone Core content of the intervention included: psychological support, knowledge about RA, treatment goals and medication adherence Based on initial assessments the nurses provided knowledge about RA and prescribed medication, reviewed progress towards achieving treatment goal emphasising the importance of self-managing the condition through medication concordance During the next 3 sessions conducted at the 4^th^, 8^th^ and 12^th^ week after hospital discharge, the nurses evaluated the adherence to their medication regimen	Rheumatology clinicians	Four individual telephone calls to the participant	Delivered over a period of 12 weeks Initially four weekly one to one sessions lasting 20–40 min The following 3 sessions were conducted at the 4^th^, 8^th^ and 12^th^ week following patient discharge	Education was tailored to patients’ specific needs identified through assessment	Not described	Intervention was delivered as planned
Liu et al. [22]	Transitional Care Programme	Patients with RA > 18 being discharged from hospital for RA related reasons	A telephone intervention The aim was to increase self-efficacy of RA patients by delivering education to empower them to manage disease activity, pain and, fatigue Patient centred assessment and goal setting to improve individual’s self-management of the disease	The intervention was divided into increasing knowledge on how to self-manage RA following hospital discharge through goalsetting, monitoring & treatment & procedures On discharge patients were telephoned within 72 h after which the nurse would provide 1–3 phone calls every week depending on the patient’s condition until 4 weeks after discharge Following the initial assessment of the patient’s health condition, the nurse focussed on the existing problems, assessing disease progression, providing advice, consolidating the patient’s self-management behaviours and health goals and encouraging patients to implement positive changes	Rheumatology specialists delivered the intervention following training in supporting self-management through telephone follow up intervention procedures	Telephone calls Participants were based in their own homes	1–3 telephone calls every week until 4 weeks after discharge With no specific restrictions on the duration of each call	Intervention was tailored according to the patients need and goals providing more phone calls as required	Not described	Intervention was delivered as planned
Zhao & Chen [23]	Self-management health education by telephone	Patients > 18 with a diagnosis of RA	Telephone health education to improve patients with RA recently discharged from hospital self-efficacy and enhance their disease management Based on the concept that effective health education can improve knowledge and influence behaviours.	Intervention was guided by a checklist which was completed to identify the patient’s educational needs regarding RA health education including information about medication, diet and exercise Clinicians delivered structured education sessions to patients over the telephone exploring patients’ beliefs and needs for health education based on their reason for hospital admission to assist them to improve self-management strategies Patients were given leaflets and verbal information about the intervention prior to discharge	Clinicians delivered the telephone educational sessions	Tele- Phone Clinicians were based at the hospital and patients in their own homes	Intervention was delivered 4 times in 12 weeks. Each call time ranged from 20–40 min	Education could be tailored to patients’ specific needs and questions	Not described	Intervention was delivered as planned
Salaffi et al. [20]	Remote TElemonitoring for MAngaing Rheumatologic Condition & HEalthcare programmes (RETE-MARCHE)	Newly diagnosed patients with RA	A telemonitoring system for patients with RA focussed on an intensive self-management strategy utilising a web-based monitoring system to achieve remission and tight disease control Based on the treat to target (T2T) strategy which promotes tight control of disease activity and aims for low disease activity or remission	RETE-MARCHE is a specialised website platform The patient completes an online RA Impact of Disease (RAID) score which measures seven domains each with a numeric rating score (NRS) Each domain has the following: weight, pain, functional disability, fatigue, sleep problems, emotional well-being, physical well-being and coping. The score has a range from 0–10 (10 worst health). The computer system generates warnings to both the patient and their clinical case manager whenever it detected based on the patients report that their condition was deteriorating. Patients were given face to face training sessions prior to using the website	Clinical case manager trained in web-based interventions	Web-based platform Participants were based in their own homes	The intervention was engaged with once a month over a period of 12 months, however patients could utilise it at any time if they felt their RA symptoms were worsening and they wanted to assess their score	Participants could utilise the platform between scheduled times if they felt their symptoms were worsening, thereby triggering follow up from the clinical case manager	Not described	Intervention was delivered as planned

Two web-based interventions [18, 19] and three telephone interventions were based on blended educational and behavioural theory [21–23]. Allam et al. [18] & Zuidema et al. [19] supported self-management through providing education and information on RA helping individuals to self-manage symptoms by targeting beliefs to affect behaviours. Allam et al. [18] incorporated a gamification feature, this approach based on self-determination theory aims to improve patient’s motivation and interaction with the website. A chat room option was available for some participants based on the theory that digital support networks may influence self-management capabilities. Behavioural theory appeared to underpin the telephone-based interventions, two were based on self-efficacy theory [22, 23] and one was based on the health belief model [21]. The main aim of these interventions was to empower patients to engage in positive self-management behaviours through planned telephone education sessions.

Two studies [20, 24] had a treat to target approach which aims to improve outcomes for patients with RA. According to NICE this is the preferred approach to provide clear direction on early treatment and tight disease control [25]. These interventions encouraged and empowered patients to be active partners in self managing their condition through online applications [24] and mobile phones [20]. These involved patients managing their condition through taking responsibility for self-monitoring of their symptoms.

### Effect of tele-health interventions on outcomes

#### Disease Activity and related symptom measures

Zuidema et al. [19] web based self-management programme found no remarkable significant effects at six months on disease activity, pain or fatigue. The outcome measurement of the RAND-36 general health perception after twelve months showed a statistically significant effect (9.65. 95% CI 0.83–18.48, *p* + 0.03) for those who used the intervention more with a small effect size 0.02. Kuusalo et al. [24] self-monitoring SMS application found no statistically significant difference in disease activity DAS 28 at 6 months despite the level of remission being higher in the intervention group. Similar DAS 28 levels were achieved in both intervention and control groups during the first 6 months, the respective mean ± SD DAS 28 scores for the intervention and control groups were 1.92 ± 1.12 and 2.22 ± 1.11 at six months (*p* = 0.09); and 1.79 + 0.91 and 2.08 + 1.22 at 12 months (*p* = 0.28). Salaffi et al. [20] trialled a web based self-monitoring application and demonstrated several findings regarding disease activity which were statistically highly significant. A higher percentage in the intervention group achieved remission (38.1% vs 25% at 12 months *p* =  < 0.01). Time to achieve remission utilising the CDAI disease activity measurement (CDAI < 2.8) was significantly shorter in the intervention group with a median of 20 weeks versus a median over 36 weeks (*p* < 0.001). The patients in the intervention group also showed a greater improvement (*p* < 0.001) in terms of functional impairment (71.4% vs 35%) and lower radiographic progression of disease than the control group (intervention vs control group 1.47 vs 2.70; *p* = 0.009). Song et al. [21] telephone based self-management support measured disease activity and found there was no statistically significant difference between in disease activity at week 12 (*p* = 0.107) and week 24 (*p* = 0.096). Zhao & Chen [23] telephone based self-management education found no significant difference in the DAS 28 score at week 12 (*p* = 0.099) and week 24 (*p* = 0.096). In contrast Liu et al. [22] telephone based self-management education for patient post hospital discharge achieved statistically significant improvements in the intervention groups HAQ-DI gripping measurement at week 8 (1.17 vs 1.46 *p* = 0.01). This may be attributed to the focus on encouraging engagement in hand-joint exercises resulting in better clinical outcomes.

### Medication adherence

Two studies looked at medication adherence as a primary outcome. Allam et al. [18] found the online self-management website did not impact medication adherence. Utilising the Prescription Medication Overuse Scale [26] a patient reported questionnaire to measure this outcome did not show any meaningful difference across the intervention groups (*p* = 0.056). Song et al. [21] telephone self-management education measured this outcome using the self-reported Medication Adherence compliance questionnaire [27] and found after the 12th week of the intervention medication adherence was significantly higher in the intervention group compared with the control group (*p* = 0.014). Similarly, the intervention group showed a significantly higher level of medication adherence than the control group at week 24 (*p* = 0.042). The effect size of the intervention on medication adherence was 0.58 (95% CI 0.12–1.03).

### Physical activity

One study measured physical activity as a primary outcome [18]. Physical activity was measured using the validated patient reported Exercise Behaviours Scale [28]. The study noted an increase in physical activity at 16 weeks in the intervention group with access to additional features social support and gaming (*p* = 0.02). There were no statistically significant outcomes found for those using the website alone.

### Health care utilisation

Allam et al. [18] evaluated the effect of a web based self-management programme on user’s health care utilisation. Utilising the validated self-reported Health Care Utilisation scale [29] to measure this primary outcome they found a significant decrease of visits to clinicians for patients accessing social support features (*p* = 0.01) and patients in accessing both social support features and gaming (*p* = 0.03). Kuusalo et al. [24] found their text-messaging to support self-monitoring application made no significant difference terms of health care utilisation. Measuring the outcome through the self-reported Utilisation of Health Care Resources scale [30] they found the number of unscheduled nurse’s visits was 0.56 + 0.80 in the intervention group and 0.56 + 0.65 in the control group (*p* = 0.56). In the intervention and control groups, the number of unscheduled physicians’ visits was 0.13 + 0.44 and 0.11 + 0.39 (*p* = 0.86) demonstrating no difference in the utilisation of healthcare resources. Liu et al. [22] measured hospital readmission scores as an outcome for the telephone self-management intervention. This did not show statistical significance although they did demonstrate clinically significant improvements as hospital readmission rates of the intervention group (4.5%) were lower than the control group (11.4%).

### Knowledge and markers of self-care

Allam et al. [18] found that patients who had access to either social support sections or the gaming experience of their website intervention scored higher on the self-reported Empowerment scale [31] at 16 weeks (p = 0.03 & p = 0.05, respectively). They also found that the web intervention did not improve RA knowledge levels, measured by the validated self-reported Patient Knowledge Questionnaire in RA [32]. This outcome did not show any significant difference between control and intervention groups (*p*-0.06). Zhao & Chen [23] found that telephone self-management education following discharge from hospital had a significant effect on self-efficacy. At baseline there was no significant difference in self-efficacy (*p* = 0.072) between the intervention group and control group. However, in the 12th and 24th week after initiating the intervention the validated patient reported Rheumatoid Arthritis Self Efficacy (RASE) [33] score was statistically highly significant (*p* =  < 0.001).

## Discussion

Five studies reported statistically significant outcomes to varying degrees [18, 20–23]. Zhao & Chen [20] and Liu et al. [22] found that tailored telephone self-management education following hospital discharge improved self-efficacy. This was measured using the patient reported outcome (PRO) Rheumatoid Arthritis Self Efficacy Score (RASE) [33]. Barlow et al. [34] endorse the RASE score to be a reliable and valid measure for people with arthritis and as a useful to evaluate self-management engagement. Liu et al. [22] assessed effectiveness over eight weeks and Zhao & Chen [23] up to 24 weeks. Further outcome measurements associated with improved self-efficacy such as medication adherence, physical activity and health care utilisation could have been considered in these studies to assess wider effects of the interventions. It would also have been of further interest to see how improvements in self-efficacy that these studies demonstrated impacted upon self-management behaviours over a longer period. Previous studies have shown self-efficacy to be a strong predictor of positive self-management behaviours among patients with other long-term conditions [35–37].

Song et al. [21] also found benefit from a tailored telephone delivered self-management support with significant improvement in medication adherence scores. This positive effect was self-reported through a validated questionnaire. Disease activity scores, however, were not improved although the effects of the intervention were only measured over the short-term 24 weeks. Longer-term effects of the intervention on other associated RA outcomes such as pain or fatigue scores could also have been considered. Chalfont et al. [38] supports that tele-health interventions focussed on improvement of patient self-efficacy and self-management may lead to improved health behaviours. These studies were implemented to support patients to self-manage on discharge from hospital which has been recognised as a vulnerable time for patients. Transitional discharge care describes self-management as a three-tiered simultaneous approach requiring knowledge, planning and ability to help patients manage their condition [39]. These findings suggest tele-health interventions may have an important role to play in supporting patients to self-manage following hospital discharge.

The tele-health interventions described in this review incorporated a range of self-management features. Findings demonstrate that tailoring interventions with multiple features or more intensive interventions may be associated with greater benefits [18, 20]. Vorderstrasse et al. [40] found that a tailored multiplatform website incorporating interactive components improved clinical, behavioural and psychosocial self-management outcomes in long-term conditions. Salaffi et al. [20] web-based intervention showed a highly significant improvement in disease activity supported by radiographic evidence over twelve months. Allam et al. [18] demonstrated that a web-based intervention incorporating gamification and social support features demonstrated significant improvements in physical activity and empowerment levels and a decrease in utilisation of health care resources. Johnson et al. [41] supports that gamification is an emerging strategy which can be beneficial to health and well-being. Further research with larger samples is required to derive meaningful conclusions on the effectiveness of these interventions to support self-management in RA patients.

Two included studies did not demonstrate any statistically significant differences on either the primary or secondary outcomes [19, 24]. Kuusalo et al. [24] tele-health intervention was based on the Treat to Target approach and promoted self-monitoring of RA symptoms [42]. Zuidema et al. [19] did not find any positive effects associated with a web-based tool to support self-management. This was surprising as the interventions were similar in both content and theory to other online self-management interventions which show positive effects in other chronic conditions [44–46]. Zuidema et al. [19] concluded that this could be due to the outcome measures chosen or perhaps the need to add a tailoring aspect to their intervention. Voncken et al. [47] supports that tailoring self-management interventions enhances patient engagement and the effectiveness of the intervention. Ammerlaan et al. [48] supports that tailoring an online RA self-management website by identifying individual goals and customising interventions improved usefulness and effectiveness.

In two studies was that participants had difficulty utilising intervention technology to undertake assessment of their own symptoms which affected their ability to participate [16, 21]. Both study authors acknowledged that they may need to redesign their interventions to be more user friendly and that patients should also be involved in the design process of future interventions. Education regarding how to use the technology was also limited and participants may have benefited from further training. Tuckson et al. [43] notes the importance of designing tele-health technology to be user friendly along with education to promote ease of access.

Overall, this review found the evidence of the effectiveness of tele-health interventions to support self-management to be inconclusive but promising. This is like the findings reported by other reviews of tele-health within other long-term conditions [49–51]. A consistent conclusion reflected in the findings of this review is that utilising tele-health interventions was not associated with worse outcomes or harm to any patient. Taking this in context it appears that whilst not consistently superior to usual care, tele-health interventions provide a safe alternative mode of delivery for supporting individuals to manage their RA. Whilst there are some promising indications of benefits that may be associated with effectiveness of the interventions it may be that sample sizes were too small to detect differences, or tools used to measure outcomes were unable to detect significant differences between groups.

## Limitations and future research

There were several limitations identified in this narrative review. Searches were limited to three databases due to time constraints and no access to an information specialist. Keywords could have been further refined to enable a more comprehensive literature search. A further limitation is that the search was also limited to studies published in English and did not look for non-English publications or unpublished literature, so it is possible that relevant studies were missed. The degree of bias within this review by only including studies published in English and the likely associated publication bias with including only published papers is acknowledged. However, the tools used in the review were rigorous and are of a high quality for both undertaking the search and extracting including the Cochrane risk of bias tool, Cochrane data extraction tool and TidIER Checklist. The methodological assessment tools used in the review are in line with the recommendations of the PRISMA statement [16].

The search identified that the studies were heterogeneous in terms of the intervention approaches, outcomes and associated contexts. The heterogeneity resulted in a further limitation as the small number of articles and associated diversity prevented the study findings being subject to a meta-analysis. The quality appraisal identified that the studies were largely deemed to be of low to moderate quality and at a high risk of bias. The sample sizes, outcome measures, short-term evaluation of interventions and locations of the studies raises further issues around the validity, reliability and generalisability of the findings from this review. Despite these limitations this review provides valuable insight into the effectiveness of tele-health interventions to support self-management in RA.

Further high-quality research is required to assess the long-term effectiveness of tele-health interventions and future trials undertaken should ensure objective outcomes are measured alongside PRO or subjective measures to increase the validity and reliability of results. Kilic et al. [52] supports that PROs are helpful in providing information to accompany clinical investigations and can be used to assist with guiding patient care, such as determining whether a clinic visit, or treatment changes are necessary. This review has highlighted for the purpose of assessing the effectiveness of tele-health interventions to support self-management that it would also be helpful if objective measures such as blood tests and radiographic evaluation were studied alongside PRO measures to support validity of findings.

The design of tele-health interventions was highlighted as an important aspect of enhancing effectiveness and patient engagement. Further research will determine the most effective behavioural and educational theories on which to base interventions. Multi-platforms and tailoring of tele-health interventions were more likely to demonstrate efficacy and interactive aspects such as social support and gamification require further exploration [18, 20]. There is growing evidence that tailoring tele-health interventions is a more successful approach and this will be an important issue to consider for future research [53]. This review also suggests that patients should be involved in the designing of tele-health interventions to promote engagement and usability. Clinicians must ensure that patients receive enough education to improve tele-health intervention literacy. Salisbury et al. [54] supports that the decision to utilise tele-health intervention to support self-management requires consensus between patient and clinicians. Further studies will help determine the wider impact of tele-health interventions on health care utilisation and associated potential benefits such as improved healthcare access and cost effectiveness.

## Conclusion

This review has highlighted that the published literature regarding the effectiveness of tele-health intervention to support self-management in RA is extremely heterogeneous. The existing evidence is limited and has not yet proven the effectiveness of tele-health interventions although there are indicators regarding its usefulness to support self-management. There are signs within this review that positive self-management outcomes are linked with tele-health interventions that are well designed, tailored and multi-faceted. Going forward tele-health interventions for individuals with RA should be patient centred, building on specific self-management theory and ensuring adequate resources are invested in education and training for users. Technologically the benefit of adding dynamic elements such as gamification to enhance interventions requires further consideration, however, simple approaches such as basic telephone interventions to support self-management have also shown to be of value in this review. Larger scale RCTs of tele-health interventions to support RA self-management are now required along with the exploration of objective validated outcomes and measurement of long-term effects. Determining the extent to which the benefits of tele-health can be harnessed to support self-management in RA will be of utmost importance as virtual care becomes increasingly utilised and especially so in the health culture of the current pandemic.

## Supplementary Information

Below is the link to the electronic supplementary material.

Supplementary file1 (DOCX 26 kb)
