# Supplementary material for: Tele-health interventions to support self-management in adults with rheumatoid arthritis: a systematic review
Source: Rheumatol Int. 2021 Jun 16;41(8):1399–418. doi: 10.1007/s00296-021-04907-2 (PMC8245362; doi:10.1007/s00296-021-04907-2)
Supplement: Supplementary file 1 — Supplementary file1 (DOCX 26 kb) [file 296_2021_4907_MOESM1_ESM.docx]

**TABLE 2 COCHRANE RISK OF BIAS TOOL [17]**

| **Selection Bias**  **Performance Bias Detection Bias Attrition Bias Reporting Bias** | | | | | | | |
| --- | --- | --- | --- | --- | --- | --- | --- |
|  | Random sequence generation | Allocation  concealment | Blinding  Patients/personnel | Blinding Outcome assessment | Incomplete data | Selective Reporting | Other biases |
| Allam et al  (2015) | High risk | Low risk | High risk | Unclear | Low risk | Unclear | Unclear |
| Zuidema et al (2019) | Unclear | Unclear | High risk | Unclear | Low risk | Low risk | Unclear |
| Kuusalo et al (2020) | High risk | Unclear | High risk | Unclear | High risk | Unclear | Unclear |
| Song et al (2019) | Unclear | Unclear | High risk | Unclear | Low risk | Unclear | High risk |
| Liu et al (2020) | Low risk | Low risk | High risk | Unclear | Low risk | Low risk | High risk |
| Zhao & Chen (2019) | Low risk | Low risk | High risk | Unclear | Low risk | Unclear | High risk |
| Salaffi et al (2019) | High risk | Low risk | High risk | Unclear | Low risk | Unclear | High risk |
